# Inhibition of Cell Proliferation and Metastasis by Scutellarein Regulating PI3K/Akt/NF-κB Signaling through PTEN Activation in Hepatocellular Carcinoma

**DOI:** 10.3390/ijms22168841

**Published:** 2021-08-17

**Authors:** Sang Eun Ha, Seong Min Kim, Preethi Vetrivel, Hun Hwan Kim, Pritam Bhagwan Bhosale, Jeong Doo Heo, Ho Jeong Lee, Gon Sup Kim

**Affiliations:** 1 Research Institute of Life Science and College of Veterinary Medicine, Gyeongsang National University, Gazwa Campus, 501 Jinju-daero, Jinju 52828, Korea; sangdis2@naver.com (S.E.H.); ksm4234@naver.com (S.M.K.); preethivetrivel05@gmail.com (P.V.); shark159753@naver.com (H.H.K.); shelake.pritam@gmail.com (P.B.B.); 2 Biological Resources Research Group, Gyeongnam Department of Environment Toxicology and Chemistry, Korea Institute of Toxicology, 17 Jegok-gil, Jinju 52834, Korea; jdher@kitox.re.kr (J.D.H.); hojeong.lee@kitox.re.kr (H.J.L.)

**Keywords:** scutellarein, proliferation, metastasis, PTEN, PI3K/Akt/NF-κB, EMT

## Abstract

Scutellarein (SCU) is a well-known flavone with a broad range of biological activities against several cancers. Human hepatocellular carcinoma (HCC) is major cancer type due to its poor prognosis even after treatment with chemotherapeutic drugs, which causes a variety of side effects in patients. Therefore, efforts have been made to develop effective biomarkers in the treatment of HCC in order to improve therapeutic outcomes using natural based agents. The current study used SCU as a treatment approach against HCC using the HepG2 cell line. Based on the cell viability assessment up to a 200 μM concentration of SCU, three low-toxic concentrations of (25, 50, and 100) μM were adopted for further investigation. SCU induced cell cycle arrest at the G2/M phase and inhibited cell migration and proliferation in HepG2 cells in a dose-dependent manner. Furthermore, increased PTEN expression by SCU led to the subsequent downregulation of PI3K/Akt/NF-κB signaling pathway related proteins. In addition, SCU regulated the metastasis with EMT and migration-related proteins in HepG2 cells. In summary, SCU inhibits cell proliferation and metastasis in HepG2 cells through PI3K/Akt/NF-κB signaling by upregulation of PTEN, suggesting that SCU might be used as a potential agent for HCC therapy.

## 1. Introduction

Liver cancer is a common malignant tumor and a leading cause of death worldwide. According to the 2020 GLOBOCAN, approximately 905,677 new cases and an estimated 830,180 liver cancer-related deaths annually, making it the sixth leading cause of cancer death, occurred worldwide in 2020. About 90% of primary liver cancers are human hepatocellular carcinoma (HCC) [1]. Despite clinical advancement, significant issues exist with the high rate of postsurgical recurrence and intrahepatic/extrahepatic metastases [2]. Current options for the treatment of HCC are chemotherapy, radiotherapy, and surgery, which largely depend upon the tumor size and stage of tumor severity. However, each therapy has serious adverse effects, while a satisfactory therapeutic effect achieved by combined therapy increases the side effects. Therefore, it is essential to find a complementary option with better efficacy and fewer side effects for HCC [3].

One of the efficient means is to discover effective biomarkers and potential therapeutic agents to increase the treatment outcome of HCC. The recent advances in selective anticancer therapeutics have mainly focused on oncogenes and tumor promoters, most often in the form of kinase inhibitors [4]. It has become more difficult to devise traditional anticancer therapeutics aimed at tumor suppressor loss. Even now, most phosphatase and tensin homolog (PTEN)-deficient cancer targeting methods use kinase inhibitors that lie at phosphatidylinositol-3 kinase (PI3K) or downstream levels [5]. In multiple tumor types, such as liver, pancreatic, breast, and bladder cancers, PTEN expression has decreased, and is highly involved in the regulation of tumor suppressors that downregulate the signaling of Akt (protein kinase B) by reducing the release of PI3K to the cell membrane [6].

Many studies have confirmed that the PI3K/Akt pathway is an important signaling pathway that regulates cell apoptosis, metabolism, proliferation, and growth [7]. In addition, in tumor development, the PI3K/Akt pathway plays a significant central role, and is closely associated with other pathways that regulate a wide range of biological processes related to tumors [8]. A lung cancer study revealed that the PI3K pathway regulated the activation of many Akt downstream substrates, such as nuclear factor-κB (NF-κB); facilitated cell proliferation, and improved chemotherapeutic drug tolerance [9]. NF-κB is one of the key molecules involved in metastasis in a wide number of molecular signaling pathways that are associated with epithelial-mesenchymal transition (EMT) [10].

EMT is a crucial tumor invasion and metastasis process. These changes increase tumor cell proliferation, and metastasis [11]. The active phosphorylated NF-κB in the nucleus will bind through chromatin to the promoter region of the Snail-1α gene, which becomes the upstream activator of Snail-1α [12]. Snail is a transcription factor that can induce the development of EMT, which in several tumors is significantly upregulated, and inhibits E-cadherin expression [13]. E-cadherin expression reduction and deletion can contribute to the disappearance of cell polarity and reduction in cell adhesion, and is an essential marker of EMT processes [14]. E-cadherin expression is negatively associated with tumor invasiveness, and epithelial cell polarity disappearance has resulted from an increase in the expression of mesenchymal cell markers N-cadherin and Snail. Thus, E-cadherin expression contributes to the occurrence of EMT processes, and promotes the initiation of metastasis [15]. Furthermore, the invasion capability of tumor cells has been strengthened. The tumor becomes vulnerable to distant metastasis, and showed an increased extent of tumor invasion [16].

In metastasis, the essential proteolytic enzymes involved in the degradation of the extracellular matrix (ECM) and basement membrane are the matrix metalloproteinases (MMPs). Their structure, function, and regulatory levels are intimately linked to play an important role in cancer metastasis [17]. Cancer chemoprevention is the use of agents that delay or inhibit the process of proliferation and metastasis, intended to reduce the possibility of invasion or clinically relevant disease development. Numerous studies have suggested that flavonoids have chemo-preventive properties [18,19].

**Figure 1:**
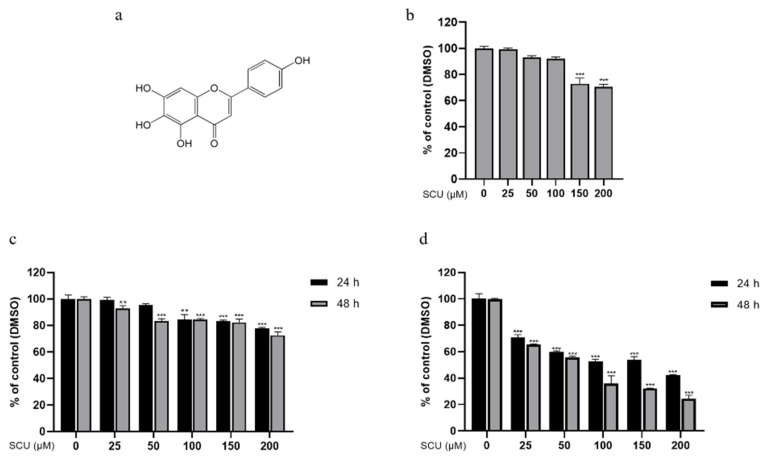
The chemical structure of scutellarein (SCU) and the effect of SCU treatment in HCC cell lines and HaCaT cells. (**a**) Structure of SCU. (**b**) Cell viability was measured by 3-(4,5-dimethylthiazol-2-yl)-2,5-diphenyltetrazolium bromide (MTT) assay with SCU (0, 25, 50, 100, 150, and 200 μM) for 48 h in HaCaT cells. (**c**) Cell viability was measured by MTT assay with SCU (0, 25, 50, 100, 150, and 200 μM) for 24 and 48 h in HepG2 cells. (**d**) Cell viability was measured by MTT assay with SCU (0, 25, 50, 100, 150, and 200 μM) for 24 and 48 h in Huh-7 cells. The results obtained from three independent experiments were expressed as mean ± standard deviation (SD) compared with the control group. ** *p* < 0.01, *** *p* < 0.001.

Scutellarein (SCU) (5,6,7,4′-tetrahydroxyflavone) (Figure 1a), a flavone found in the perennial herb *Scutellaria baicalensis*, has a wide range of biological functions [20]. The elevated bioavailability of SCU has been attributed to its enhanced solubility, when compared to scutellarin (40,5,6-trihydroxyfl avone-7-glucuronide). In addition, SCU is absorbed mainly in the form of its hydrolyzed counterpart, which is more active than scutellarin [21]. Reported studies demonstrated the significant anticancer effect of SCU in HCC cell lines [22,23]. In this study, we investigate the cell proliferation and metastasis inhibitory effects on SCU and their underlying molecular mechanism in HepG2 cells. Thus, our study suggests flavonoid SCU can be potentially used for its anticancer effect through the inhibition of the PI3K/Akt/NF-κB signaling pathway by the upregulation of PTEN in HepG2 cells.

## 2. Results

### 2.1. Scutellarein (SCU) Inhibits Cell Viability in Human Hepatocellular Carcinoma (HCC) Cell Lines and HaCaT Cells

SCU treatment in human normal keratinocyte HaCaT cells did not affect at 25, 50, and 100 μM for 48 h (Figure 1b). HCC cell lines HepG2 and Huh-7 cells were treated with or without different concentrations of SCU of (0, 25, 50, 100, 150, and 200) μM for (24 and 48) h. Figure 1c, shows that SCU exhibited almost no cell death in HepG2 cells. Whereas, in Huh-7 cells significantly decreased the cell viability in a dose and time dependent manner (Figure 1d). These results suggest that concentrations of (25, 50, and 100) μM at 48 h were of low toxicity in HepG2 cells, therefore those doses and time were used for subsequent experiments.

### 2.2. SCU Inhibits Cell Migration and Proliferation in HepG2 Cells

**Figure 2:**
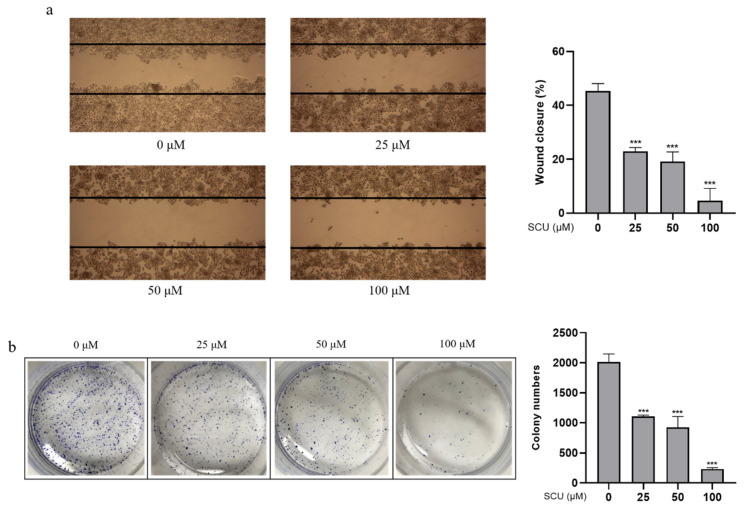
Effect of SCU on HepG2 cell migration and proliferation. (**a**) Wound-healing assay measured the migration ability changes. Cells were treated with SCU (0, 25, 50, and 100 μM) for 48 h. (**b**) Colony formation assay measured antiproliferation effect. Cells were treated with SCU (0, 25, 50, and 100 μM) for 2 weeks. The results obtain from three independent experiments were expressed as mean ± standard deviation (SD) compared with the control group. *** *p* < 0.001.

To confirm the inhibitory effects of SCU, cells were treated with the indicated concentrations of (0, 25, 50, and 100) μM for 48 h. Inhibition of cell migration was confirmed by wound healing assay. Figure 2a shows that SCU significantly inhibited the cell migration of marginal cells in a concentration-dependent manner. Similarly, the results of cell proliferation were measured by colony formation assay, which showed that SCU significantly decreased the number of colonies in a concentration-dependent manner (Figure 2b).

### 2.3. SCU Induces G2/M Phase Cell Cycle Arrest in HepG2 Cells

**Figure 3:**
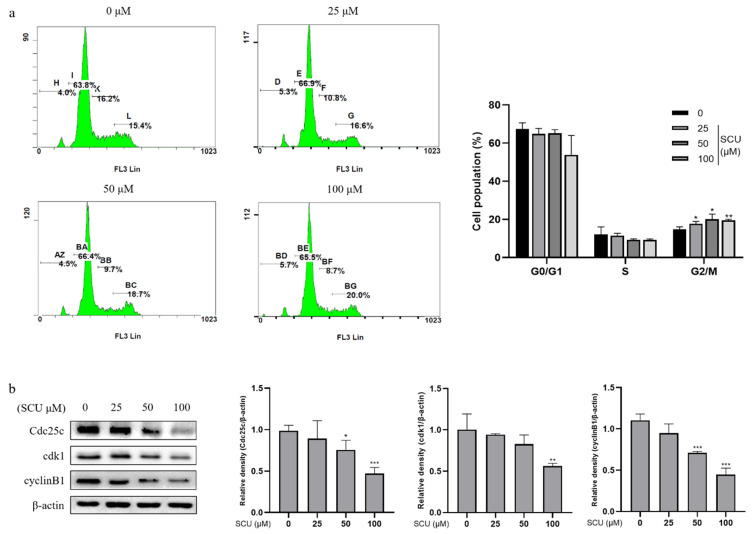
Effect of SCU on cell cycle progression in HepG2 cells. (**a**) SCU induces HepG2 cell G2/M cell cycle arrest. Cells were treated with SCU (0, 25, 50, and 100 μM) for 48 h and the cell cycle was detected by flow cytometry. (**b**) Cdc25c, cdk1, and cyclin B1 levels were quantified. The results obtained from three independent experiments were expressed as mean ± standard deviation (SD) compared with the control group. * *p* < 0.05, ** *p* < 0.01, *** *p* < 0.001.

To investigate the mechanism of cell cycle arrest by SCU in HepG2 cells we used flow cytometry analysis. The cells were treated with SCU at the indicated concentrations of (0, 25, 50, and 100) μM for 48 h, followed by staining with propidium iodide (PI) and cell cycle distribution analysis. Figure 3a shows that SCU increased the percentage of G2/M phase fraction. Further, we measured the expression levels of the G2/M-related proteins by immunoblotting. From this, it was found that SCU induced G2/M phase arrest in HepG2 cells (Figure 3b).

### 2.4. SCU Induces PTEN Expression in HepG2 Cells and Validation of SCU Binding with PTEN Using In Silico Molecular Docking Analysis

**Figure 4:**
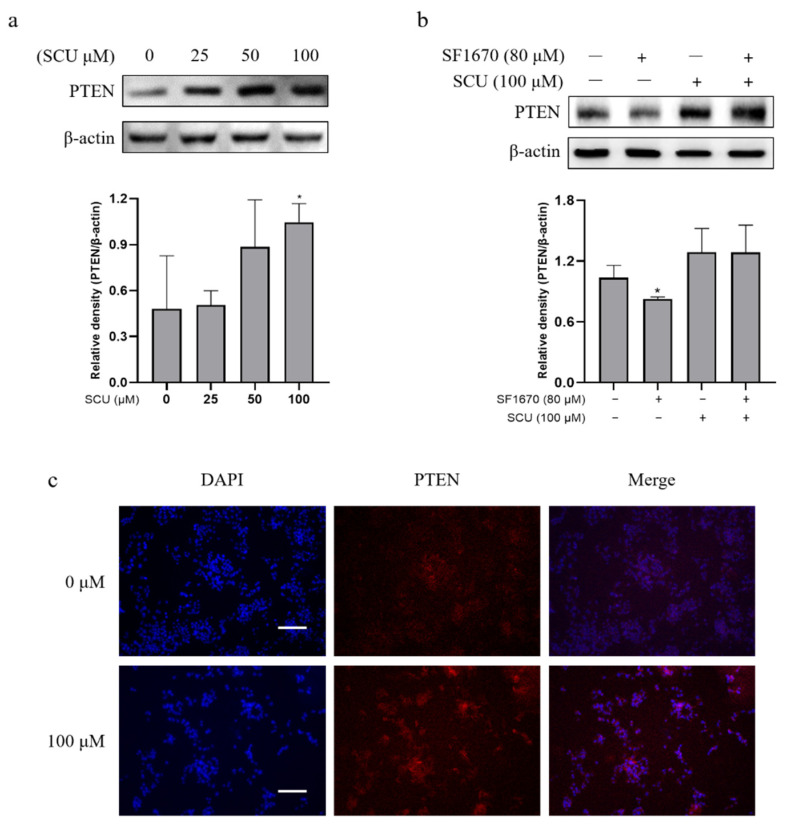
Effect of SCU on PTEN expression in HepG2 cells. (**a**) SCU induces PTEN protein level in HepG2 cells. Cells were treated with SCU (0, 25, 50, and 100 μM) for 48 h. (**b**) Effect of PTEN inhibitor and SCU-induced PTEN level in HepG2 cells. Cells were treated with or without 80 μM of PTEN inhibitor: SF1670 and 100 μM of SCU for 48 h. (**c**) Representative immunofluorescence image images of expression of PTEN were evaluated at 0 and 100 μM (scale bars = 100 μm). The results obtained from three independent experiments were expressed as mean ± standard deviation (SD) compared with the control group. * *p* < 0.05.

**Figure 5:**
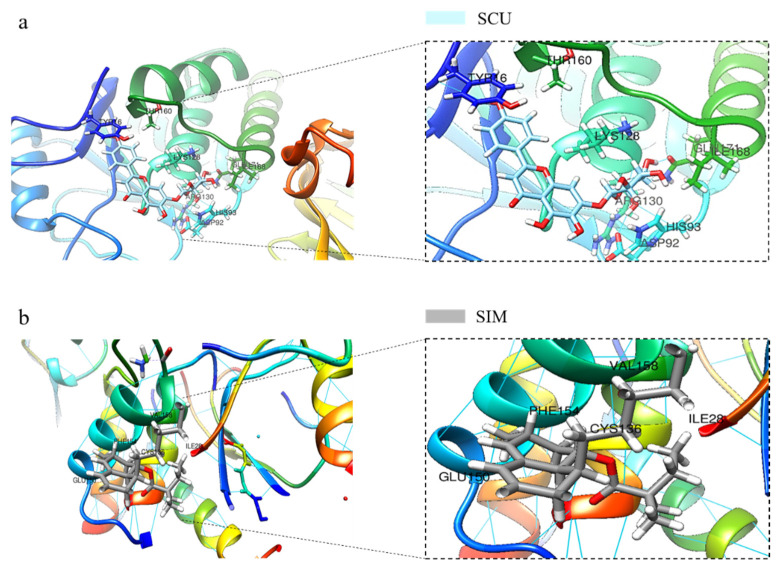
In silico molecular docking analysis of the ligands SCU and simvastatin (SIM) with target PTEN. (**a**) The 3D structure of PTEN bound efficiently with compound SCU with its interacting amino acids TYR16, THR160, ASP92, HIS93, ARG130, LYS128, GLN171, and ILE168. (**b**) The 3D structure of PTEN bound efficiently with the known activator compound SIM with its interacting amino acids ILE28, CYS136, PHE154, GLU150, and VAL158.

To determine whether the induction of PTEN was associated with the treatment of SCU and PTEN, the protein level was analyzed by immunoblotting. The cells were treated with SCU at the indicated concentrations of (0, 25, 50, and 100) μM for 48 h. Figure 4a shows that SCU-treated cells significantly increased the expression of PTEN. Several proteins have been involved in the negative regulation of PI3K activation [24]. Among these, PTEN is the most crucial negative regulator of the PI3K signaling pathway [25]. Therefore, to confirm the activation of PTEN by SCU in HepG2 cells, we performed an inhibitor assay. The cells were treated with or without 80 μM of PTEN inhibitor SF1670 and 100 μM of SCU for 48 h. The results revealed that SCU significantly upregulated the protein expression of PTEN, and the activity of SCU was higher than that of the PTEN inhibitor (Figure 4b). Consistently, immunofluorescence assay also demonstrated the markedly increased expression of PTEN due to SCU treatment (Figure 4c). To support the interaction of PTEN with SCU, we performed in silico molecular docking, and compared the binding affinity of the complex to a reference PTEN activator simvastatin (SIM). The protein structure of PTEN with the ligands SCU and SIM was subjected to ligand protein docking using UCSF Chimera software (https://www.cgl.ucsf.edu/chimera/, accessed on 10 August 2021). Figure 5a,b represent the results, which show the bound complex of SCU and SIM with PTEN. The molecular dock score revealed the binding affinity with an estimated free energy of −6.5 kcal/mol by SIM, followed by comparably higher free energy of −8.4 kcal/mol by SCU. In addition, the interacting amino acid residues involved in the bound complex of PTEN with SCU were found to be TYR16, THR160, ASP92, HIS93, ARG130, LYS128, GLN171, and ILE168. Whereas the PTEN with SIM complex includes ILE28, CYS136, PHE154, GLU150, and VAL158. The obtained results of docking suggest that SCU shows a higher binding affinity with PTEN, compared to the reference activator SIM. Overall, our findings indicate that SCU as a PTEN activator and may be useful in treatment option in HCC.

### 2.5. SCU Suppresses the PI3K/Akt/NF-κB Signaling Pathway in HepG2 Cells

**Figure 6:**
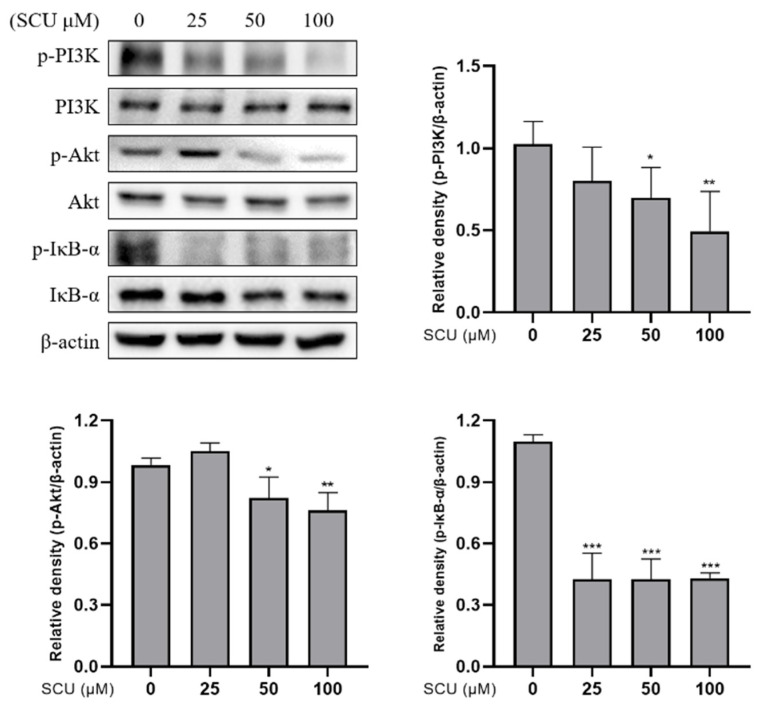
Effect of SCU on PI3K/Akt/NF-κB signaling pathway in HepG2 cells. SCU suppresses p-PI3K, p-Akt, and p-IκB-α levels in HepG2 cells. Cells were treated with SCU (0, 25, 50, and 100 μM) for 48 h. The results obtained from three independent experiments were expressed as mean ± standard deviation (SD) compared with the control group. * *p* < 0.05, ** *p* < 0.01, *** *p* < 0.001.

**Figure 7:**
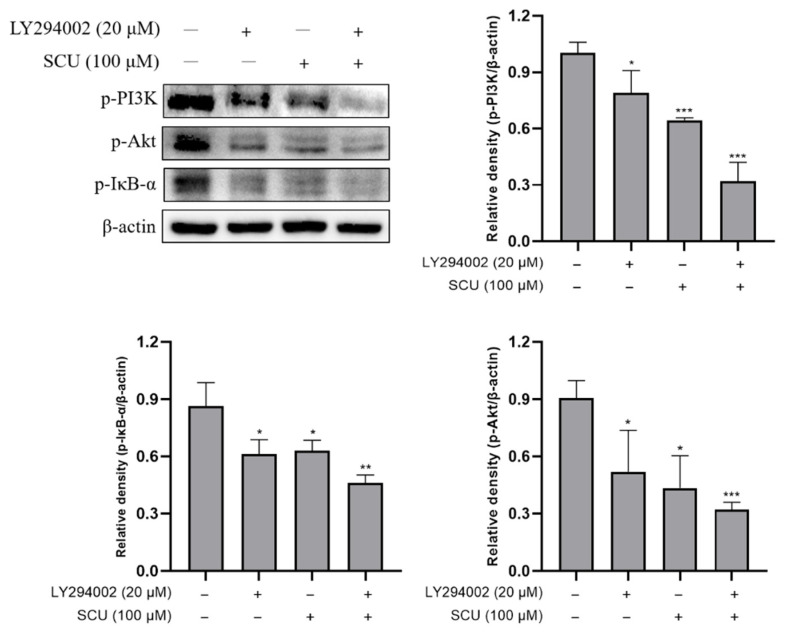
Inhibitory effect on PI3K/Akt/NF-κB signaling pathway in HepG2 cells. Effect of PI3K inhibitor and SCU-induced inhibition of p-PI3K, p-Akt, and p-IκB-α levels in HepG2 cells. Cells were treated with or without 20 μM of PI3K inhibitor: LY294002 and 100 μM of SCU for 48 h. The results obtained from three independent experiments were expressed as mean ± standard deviation (SD) compared with the control group. * *p* < 0.05, ** *p* < 0.01, *** *p* < 0.001.

To investigate whether the PI3K/Akt/NF-κB pathway was involved in the inhibition of cell proliferation effects of SCU, the cells were treated with SCU at the indicated concentrations of (0, 25, 50, and 100) μM for 48 h. The immunoblotting data showed that the SCU significantly downregulated protein expressions of p-PI3K, p-Akt, and p-IκB-α (Figure 6). In addition, to confirm the effect of SCU on PI3K inhibition, examinations were performed with or without 20 μM of PI3K inhibitor: LY294002 and 100 μM of SCU for 48 h. The result revealed that SCU significantly downregulated the protein expressions of p-PI3K, p-Akt, and p-IκB-α similar to LY294002 (Figure 7). Taken together, these data suggest that SCU suppresses the PI3K/Akt/NF-κB signaling pathway in HepG2 cells.

### 2.6. SCU Inhibits the Activity of EMT Markers and Migration-Related Proteins in HepG2 Cells

**Figure 8:**
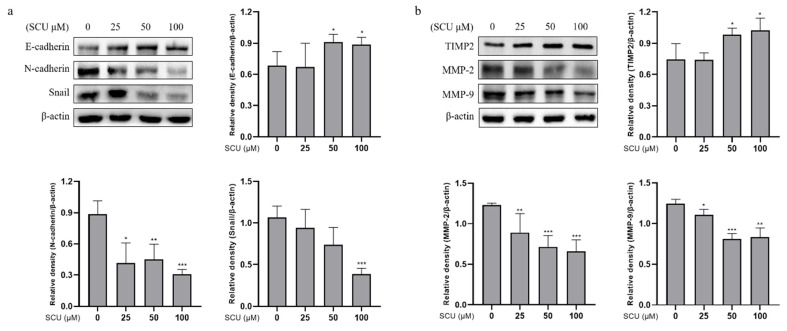
Effect of SCU on EMT markers and migration-related proteins in HepG2 cells. (**a**) SCU induces EMT-related proteins E-cadherin and suppresses N-cadherin and Snail levels in HepG2 cells. Cells were treated with SCU (0, 25, 50, and 100 μM) for 48 h. (**b**) SCU induces extracellular inhibitor of MMPs: TIMP2 and suppresses migration-related proteins MMP-2 and MMP-9 levels in HepG2 cells. The results obtained from three independent experiments were expressed as mean ± standard deviation (SD) compared with the control group. * *p* < 0.05, ** *p* < 0.01, *** *p* < 0.001.

To verify the migration and invasion inhibitory effect of SCU, we performed experiments to investigate the epithelial-to-mesenchymal transition (EMT) markers and migration-related proteins. We assessed the expression levels of EMT-related proteins (E-cadherin, N-cadherin, and Snail) and migration-related proteins (TIMP2, MMP-2, and MMP-9) by immunoblotting. The cells were treated with SCU at the indicated concentrations of (0, 25, 50, and 100) μM for 48 h. Figure 8a shows that SCU upregulated the expression of E-cadherin, whereas N-cadherin and Snail were downregulated, respectively. Moreover, SCU induced the extracellular inhibitor of MMPs: TIMP2, but downregulated MMP-2 and MMP-9 in a dose-dependent manner (Figure 8b). These findings indicate that SCU inhibits HepG2 cell metastasis through the regulation of EMT markers and migration-related proteins.

## 3. Discussion

Chemotherapy represents the world’s oldest method of medicine. Two-thirds of the world’s population were estimated to resort to medicinal plants originating from folk medicine [26]. There are at least 250,000 plant species, of which more than 1000 plants have been found to have effective anticancer properties [27]. Representatively, plant-derived compounds, such as vincristine, irinotecan, etoposide, and paclitaxel, are being used to treat cancer [28]. While several molecules acquired from nature have worked wonders, there are numerous molecules that medicinal chemists still need to trap or analyze in depth. In the treatment of cancer, plant-based drugs have also found important roles, and the mechanism of association between diverse phytochemicals and cancer cells is being extensively studied [27]. The antiproliferation effect of one compound is linked to several variables, which include cell type, cell sensitivity, cell density seeded in the plates, time of treatment, and concentration of reagents used in the experiment [29].

In this study, we first confirmed the antimigration and antiproliferation effect of scutellarein (SCU) on human hepatocellular carcinoma (HCC) cells. SCU significantly suppressed the cell migration and proliferation in HepG2 cells. The fate of normal liver tissue depends upon the balance between replication and apoptosis. In normal liver tissue, the balance between cell replication and apoptosis is maintained; however, in precancerous and cancerous tissues, this balance is disrupted [30]. Furthermore, the cell cycle transition is a potential target for cancer diagnosis and treatment [31]. In this context, we explored whether SCU was able to affect cell proliferation by targeting the cyclin and cyclin-dependent kinase (cdk) that regulates each phase of the cell cycle, as another potential function of the preventive action of the flavonoid [32]. The findings showed that SCU induced an accumulation of G2/M phase fraction and decreased the related proteins, indicating a major impact on G2/M arrest. Mitosis initiation is regulated by the activation of the cyclin B/cdk1 complex, as is widely recognized, so decreased levels of cyclin B1 protein will induce G2 arrest, and the inhibition of cyclin B1 transcription triggers cell cycle arrest [33]. In addition, Cdc2/cyclin B was the first kinase reported to phosphorylate and activate Cdc25c in vitro [34].

Recent development has mostly focused on oncogenes and tumor promoters, most generally in the form of kinase inhibitors, in selective anticancer therapeutic strategies. Traditionally, the development of anticancer therapy aimed at the loss of tumor suppressors has become more complex. Phosphatase and tensin homolog (PTEN) lead to tumor suppression, emphasizing the intricacy of strategies to therapeutically target PTEN-deficient tumors. For the treatment of patients with PTEN-deficient tumors, several medications are currently under clinical trials [35]. Knowledge of drug sensitivity gained from preclinical trials has been partly validated in the clinic. The important tumor suppressing properties of PTEN are as a lipid phosphatase, which antagonizes phosphatidylinositol 3-kinase signaling (PI3K) [36]. Even now, most PTEN-deficient cancer targeting strategies use kinase inhibitors that lie at PI3K or downstream levels [5].

In a major signaling cascade, PI3K is a crucial node that controls cancer cell growth, survival, and metabolism. In addition, intracellular localization plays a significant role in controlling the function of PTEN [37]. Several research results have suggested that the PI3K/Akt (protein kinase B) pathway plays an important role in the regulation of cancer cell migration. Previous studies revealed that the PI3K/Akt pathway can upregulate the secretion of metalloproteinase (MMP)-2 and -9 to enhance the invasion ability of ovarian cancer cells [38]. According to previous studies, the Akt pathway promotes the migration of cancer cells via the activation of nuclear factor-κB (NF-κB) by phosphorylating IκB kinase [39].

In certain cancers, and as a result of chemotherapy, the transcription factor NF-κB is activated. In the transcriptional activation of genes linked with proliferation, the central role of NF-κB tends to be metastasis to promote oncogenesis and cancer therapy resistance. Nevertheless, the existing data are sufficiently promising to consider careful research to dissect the role of NF-κB in a number of cancers, and to assess the applicability of NF-κB inhibition as an adjuvant strategy in conventional cancer therapy approaches [40]. Collectively, our findings suggest that SCU upregulates PTEN, and consequently downregulates PI3K/Akt and IκB-α expression. Inhibitor assay with PTEN inhibitor SF1670 supports the suggestion that SCU is a PTEN activator. To the best of our knowledge, PTEN activation in SCU treated HepG2 cells plays a significant role. In addition, we used LY294002, a specific inhibitor of PI3K, to confirm whether SCU treatment could inhibit the PI3K signaling pathways, similar to LY294002. LY294002 is a synthetic PI3K inhibitor based on quercetin, a flavonoid found in nature that inhibits a wide range of protein kinases [41]. Treatment with SCU significantly downregulated the expression of PI3K and its downstream proteins.

Cancer metastasis is a complex process involving several executions of downstream cellular processes, as is well known. Epithelial–mesenchymal transition (EMT) is the central cellular process for the progression of metastasis that enables tumor cell migration, invasion, etc. It could also be a significant tumor growth mechanism [38]. In addition, the intracellular signaling mediators activated by mitogens were involved in positive regulation of the EMT processes. During EMT, migration, invasion, and adhesion involve the switch of the immobile epithelial-like cancer cells to a motile mesenchymal-like phenotype [42]. Transcription factors, such as Snail, Slug, and NF-κB have been reported to play important roles in controlling EMT. Moreover, Snail is capable of downregulating E-cadherin expression by binding to E-boxes in the promoter of E-cadherin [43]. As previous studies have also shown, the molecular mechanism underlying PI3K/Akt mediated Snail inhibition through GSK-3β, which is a downstream effector of Akt [44]. Further, several studies have also shown it to be controlled by NF-kB, as discussed [45]. Our results also showed that SCU could markedly upregulate the E-cadherin but downregulate the N-cadherin and Snail protein expressions.

**Figure 9:**
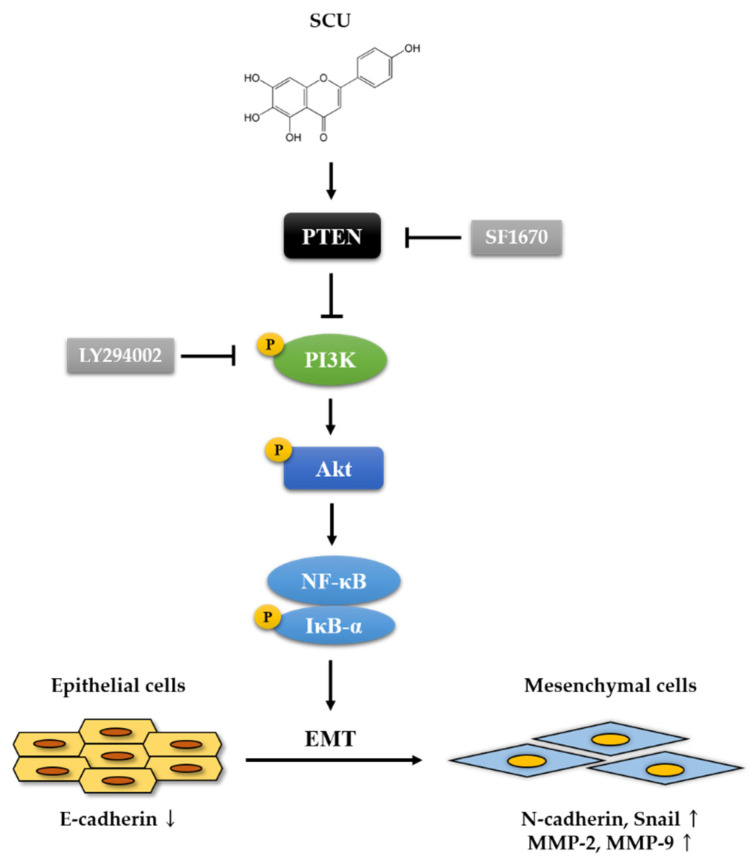
Schematic representation of the inhibition of HepG2 cell proliferation and metastasis by SCU. SCU inhibits proliferation and metastasis through suppression of PI3K/Akt/NF-κB signaling pathway by inducing PTEN and regulating EMT markers and migration-related proteins (E-cadherin, N-cadherin, Snail, TIMP2, MMP-2, and MMP-9) in HepG2 cells.

MMPs are regulated at various levels, including transcriptional and posttranslational mechanisms. MMPs are dependent on inhibition by both the nonspecific proteinase inhibitor alpha2-macroglobulin, and specifically, by the tissue inhibitors of metalloproteinase (TIMP). TIMP-1, TIMP-2, TIMP-3, and TIMP-4 are the four gene products currently identified in the TIMP family. TIMPs inactivate MMPs by binding to their active sites, and forming stable MMP–TIMP complexes [46]. In the follow-up exploration, we investigated the migration-related proteins, such as MMP-2, MMP-9, and TIMP2, a MMPs inhibitor. According to our results, SCU is a potent regulator of the EMT processes. Taken together, our current study shows that SCU could inhibit the PI3K/Akt/NF-κB pathway by upregulating the expression of PTEN in vitro, which might explain how SCU inhibits cell proliferation and metastasis in HepG2 cells (Figure 9).

## 4. Materials and Methods

### 4.1. Chemicals and Reagents

Scutellarein (SCU) was purchased from Chengdu Biopurify Phytochemicals Ltd. (purity: >98%, Chengdu, Sichuan, China). Human hepatocellular carcinoma (HCC) cell lines HepG2, Huh-7, and human normal keratinocyte cell line HaCaT were cultured in Dulbecco’s modified Eagle’s medium (DMEM). DMEM, fetal bovine serum (FBS), phosphate-buffered saline (PBS), and antibiotics penicillin/streptomycin (P/S) were purchased from Gibco (BRL Life Technologies, Grand Island, NY, USA). 3-(4,5-Dimethylthiazol-2-yl)-2,5-diphenyltetrazolium bromide (MTT) was purchased from Duchefa Biochemie (Haarlem, Netherlands). Giemsa stain was purchased from Sigma-Aldrich Co. (St. Louis, MO, USA). Antibodies to Cdc25c (cat. No. 4688S), cyclin B1 (cat. No. 4138S), β-actin (cat. No. 4970S), PTEN (cat. No. 9559S), Phospho-PI3K (cat. No. 4228S), PI3K (cat. No. 3011S), Phospho-Akt (cat. No. 4060S), Akt (cat. No. 2920S), Phospho-IκB-α (cat. No. 2859S), IκB-α (cat. No. 4812S), LY294002 (cat. No. 9901S), and Snail (cat. No. 3879S) were purchased from Cell Signaling Technology (Danvers, MA, USA). Antibodies cdk1 (cat. No. ab131450), SF1670 (cat. No. ab141303), E-cadherin (cat. No. ab1416), N-cadherin (cat. No. ab76011), TIMP2 (cat. No. ab180630), MMP-2 (cat. No. ab97779), and MMP-9 (cat. No. ab38893) were obtained from Abcam (Cambridge, England). Horseradish peroxidase (HRP)-conjugated secondary antibodies to antirabbit (cat. No. A120-101P) and antimouse (cat. No. A90-116P) were obtained from Bethyl Laboratories, Inc. (Montgomery, AL, USA).

### 4.2. Cell Culture and SCU Treatment

HepG2 cells were cultured in DMEM medium containing 10% fetal bovine serum (FBS), and 1% penicillin/streptomycin (P/S) at 37 °C in a humidified atmosphere of 5% CO_2_. SCU was prepared with dimethyl sulfoxide (DMSO), stored at −20 °C, and diluted to the required concentration with DMEM medium before use. Cells were DMSO-treated or -untreated with the indicated concentration of SCU for 48 h in a complete medium and in all experiments, final concentration of DMSO was used below 0.1%.

### 4.3. Cell Viability Assay

Cell viability was measured using MTT assay. Initially, MTT assay was carried out, for which HCC HepG2 and Huh-7 cells were seeded into 48-well plates at 5 × 10^4^ cells per well, followed by treatment at various concentrations of SCU of (0, 25, 50, 100, 150, and 200) μM for (24 and 48) h. Human normal keratinocyte HaCaT cells were seeded into 48-well plates at 5 × 10^4^ cells per well, followed by treatment at various concentrations of SCU of (0, 25, 50, 100, 150, and 200) μM for 48 h. After incubation, 50 μL of MTT (0.5 mg/mL) solution was added to each well, and incubated for about 3 h at 37 °C. The formazan precipitate formed after incubation was dissolved in 300 μL of DMSO, and the absorbance of the converted dye was measured at a wavelength of 450 nm by microplate reader (BioTek, Winooski, VT, USA). Cell viability was expressed as a percentage of proliferation versus the SCU-untreated group.

### 4.4. Wound Healing Assay

HepG2 cells were seeded into 6-well plates at 6 × 10^5^ cells per well, incubated overnight to form a confluent cell monolayer, and crossed vertically with a yellow tip in the middle of each well. Plates were then washed with PBS to remove the scraped cells. After treatment with the indicated concentrations of SCU of (0, 25, 50, and 100) μM, they were incubated for 48 h. The images of the scratches were obtained under microscopy.

### 4.5. Colony Formation Assay

HepG2 cells were seeded into 6-well plates at 1 × 10^4^ cells per well. The cells were treated with the indicated concentrations of SCU of (0, 25, 50, and 100) μM, followed by incubation for 14 days. After incubation, the grown colonies were fixed with 4% paraformaldehyde for 30 min, then stained with 0.6% Giemsa stain for 30 min, and further washed with tap water to remove excess stain. The obtained colonies were counted using the ImageJ software program (U.S. National Institutes of Health, Bethesda, MD, USA).

### 4.6. Analysis of Cell Cycle Distribution by Flow Cytometry

Flow cytometry was performed to analyze the cell cycle distribution and cell death. HepG2 cells were seeded into 60 mm plates at 5 × 10^5^ cells per well, and treated with the indicated concentrations of SCU of (0, 25, 50, and 100) μM for 48 h. After incubation, cells were collected, and fixed in 70% ice-cold ethanol for 1 h at −20 °C. The cells were washed in ice-cold PBS once, then resuspended in 500 μL of PBS containing 50 μg/mL propidium iodide (PI) (Sigma-Aldrich, St. Louis, MO, USA) and 50 μg/mL RNase A, followed by incubation in dark conditions for 15 min at room temperature (RT). The cells were immediately analyzed by flow cytometry with Cytomics FC 500 (Beckman Coulter, Brea, CA, USA). In each sample, approximately 10,000 cells were sorted. The data obtained were analyzed using CXP Software (Beckman Coulter, Inc., Fullerton, CA, USA).

### 4.7. Western Blot Analysis

HepG2 cells were seeded into 60 mm plates at 5 × 10^5^ cells per well, and treated with the indicated concentrations of SCU of (0, 25, 50, and 100) μM for 48 h. Cells were lysed using radioimmunoprecipitation assay (RIPA) buffer (iNtRON Biotechnology, Seoul, Korea) containing phosphatase and protease inhibitor cocktail (Thermo Scientific, Rockford, IL, USA). Protein concentrations were determined using a Pierce™ BCA assay (Thermo Fisher Scientific, Rockford, IL, USA). An equal quantity of protein (10 μg) from each sample was electrophoresed on (8–15)% SDS-polyacrylamide gels, and transferred to a polyvinylidene difluoride (PVDF) membrane (ATTO Co., Ltd., Tokyo, Japan), and then the membrane was incubated with the primary antibodies followed by a conjugated secondary antibody to peroxidase. The obtained proteins were detected by electrochemiluminescence (ECL) detection system (Bio-Rad Laboratory, Hercules, CA, USA), and analyzed using the Image Lab 4.1 (Bio-Rad) program. The densitometry readings of the protein bands were normalized by comparison with the expression of β-actin as control, using the ImageJ software program (U.S. National Institutes of Health, Bethesda, MD, USA).

### 4.8. Inhibitor Assay

To investigate the effect of PI3K as an upstream target of PI3K/Akt/NF-κB signaling pathway on the inhibition of cell proliferation in HepG2 cells by SCU, an inhibitor assay was performed. HepG2 cells were seeded into 60 mm plates at 5 × 10^5^ cells per well, and treated with 100 μM of SCU, along with cotreatment of inhibitor LY294002 (a PI3K specific inhibitor) at 20 μM, followed by 48 h incubation. The protein expressions of p-PI3K, p-Akt, and p-IκB-α were quantified using Western blot, as described above. To investigate the activation of PTEN as an important negative regulator of the PI3K signaling pathway in HepG2 cells by SCU, an inhibitor assay was performed. HepG2 cells were seeded into 60 mm plates at 5 × 10^5^ cells per well, and treated with 100 μM of SCU, along with co-treatment of inhibitor SF1670 (a PTEN inhibitor) at 80 μM, followed by 48 h incubation. The protein expression of PTEN was quantified using Western blot, as described above. The densitometry readings of the protein bands were normalized by comparison with the expression of β-actin as control.

### 4.9. Immunocytochemistry

HepG2 cells were seeded into 6 well plates at 3 × 10^5^ cells per well, and treated with 100 μM of SCU for 48 h. Cultured cells were washed with PBS, and fixed in 4% paraformaldehyde for 10 min. Fixed cells were further washed with PBS, and immersed in 5% normal goat serum for 1 h at RT to block nonspecific reaction. Subsequently, the cells were incubated with anti-PTEN (cat. No. ab170941, Abcam, Cambridge, England) overnight at 4 °C. Cells were washed with PBS, and reacted with fluorescein isothiocyanate (FITC)-labeled goat antirabbit secondary antibody (cat. No. 8889S, Cell Signaling Technology, Danvers, MA, USA) for 2 h. Cells were then washed with PBS, and coverslipped with DAPI (Vector Laboratories, Burlingame, CA, USA) for 10 min. PTEN-positive cells were observed under fluorescence microscopy (Nikon Corporation Ts2 FL, Tokyo, Japan). PTEN-positive cell values were evaluated with the percentage of the number of PTEN-positive cells to the number of DAPI-positive cells.

### 4.10. Molecular Docking Studies

For the execution of molecular docking, the structure of PTEN was obtained from a protein data bank (PDB) (https://www.rcsb.org/, accessed on 17 April 2021) with PDB ID 1D5R at high resolution, and the three-dimensional structures of the compound SCU and simvastatin (SIM) were obtained from PubChem (https://pubchem.ncbi.nlm.nih.gov/, accessed on 17 April 2021). The protein and ligand were subjected to docking using the USCF Chimera program, and all the possible conformations were obtained with default parameters. The results were evaluated based on the estimated free energy of binding and total intermolecular energy.

### 4.11. Statistical Analysis

All experimental results were expressed as the mean ± standard deviation (SD) of triplicate samples. Significant differences between groups were calculated by one-way factorial analysis of variance (ANOVA), followed by a Student’s test, and *p* < 0.05 was considered statistically significant. * *p* < 0.05, ** *p* < 0.01, *** *p* < 0.001.

## 5. Conclusions

In conclusion, SCU inhibits cell proliferation and metastasis through the PI3K/Akt/NF-κB signaling pathway by upregulating PTEN in HepG2 cells. This study also provides evidence of the SCU playing a significant role as a potent activator of PTEN and inhibitor of PI3K signaling pathways in HepG2 cells. The obtained results may provide new insight into the molecular mechanism of SCU in HepG2, and may have potential therapeutic strategies for HCC. However, the present study has minor limitations with the use of only one HCC cell line, and clinical investigations are warranted for immediate human application.

## Data Availability

Not applicable.
